# Lecithin’s Roles in Oleogelation

**DOI:** 10.3390/gels10030169

**Published:** 2024-02-27

**Authors:** Joanna Harasym, Karol Banaś

**Affiliations:** 1 Department of Biotechnology and Food Analysis, Wroclaw University of Economics and Business, Komandorska 118/120, 53-345 Wroclaw, Poland; karol.banas@ue.wroc.pl; 2 Adaptive Food Systems Accelerator-Science Centre, Wroclaw University of Economics and Business, Komandorska 118/120, 53-345 Wroclaw, Poland

**Keywords:** lecithin, oleogel, structure, texture, functionality, nutrition

## Abstract

This manuscript analyzes the research considering the exploitation of lecithin in oleogelation. The main objective of the work was to gather, analyze, and extract from the existing research data the information that enables us to identify lecithin-dependent roles. Oleogelation is still under research, while using various oleogelators and structurants provides changes on different physico-chemical levels. Multivariable formulations do not facilitate the elucidation of the specific role of any of them. Lecithin, due to its complex structure, big molecule, and amphiphilic nature, can provide different functionalities in complex matrices like oleogels. Therefore, this review identifies and categorizes the functionality of lecithin in oleogelation into four main roles: 1. oleogelation facilitator; 2. structure-forming impact; 3. texturing agent; and 4. functionality provider. Also, the origin and structure-forming characteristics of lecithin, as well as a short summary of the oleogelation process itself, are presented. Our critical analysis allowed us to identify the roles of lecithin in the oleogelation process and categorized them as follows: oleogelator, emulsifier, structural organization facilitator, structural modifier, crystal characteristics modifier, self-assembly promoter, thermal behavior changer, hydrogen-bonded networks promoter, hydrogel structure modifier, texture and structural modifier, gel-like state promoter, oil capacity enhancer, functionality provider, shelf life extender, and bioavailability and bioaccessibility enhancer. Lecithin came out as an important and multifunctional compound whose applications in oleogelation need to be thoroughly pre-considered. It is crucial to grasp all the possible roles of used compounds to be able to predict the final functionality and characteristics of formed oleogel matrices.

## 1. Introduction

Lecithin is a lipid molecule that is a key component of all living cells and has a unique molecular structure that allows it to perform various functions, which makes it widely used in pharmaceuticals, cosmetics, and food industries due to its emulsifying, viscosity modifying, stabilizing, solubilizing, and penetration-enhancing properties [1]. It consists of a complex mixture of acetone-insoluble phosphatides, primarily consisting of phosphatidyl choline, phosphatidyl ethanolamine, phosphatidyl serine, and phosphatidyl inositol, along with other substances such as triglycerides and fatty acids [1,2].

Lecithin molecules arrange themselves at the boundary between immiscible liquids such as oil and water, reducing the interfacial tension and forming relatively stable emulsions. However, poorly purified or synthetic lecithins do not exhibit gelation properties [1]. Lecithins can be derived from various sources, including vegetables and land and marine animal sources, and their composition and physical characteristics can vary depending on the origin and extraction process [3,4]. In the context of food and industry, lecithin is a versatile and valuable byproduct of the oilseed industry. It serves multiple functions, including emulsification, wetting, viscosity reduction, acting as a release agent, crystallization control, and more [5]. 

Lecithin is commonly sourced from oil-bearing seeds such as soybeans, rapeseeds, and sunflower kernels, with soybean being the most studied source [3,4,5,6,7]. Animal sources, including egg yolk and marine animals, also provide lecithin, with egg yolk having a high content and marine animals being noted for their high phospholipid composition [3,7]. By-products from the soybean oil industry, such as acidulated soy soapstock and deoiled soy lecithin, are valuable sources of lecithin for poultry feeds and other applications [2,8].

Modified lecithins are structurally diverse phospholipids that have been altered to enhance their functional properties, such as emulsification, dispersibility, and stability in various applications. These modifications are achieved through physical, chemical, and enzymatic methods, tailoring lecithins to specific industrial needs. Modified lecithins exhibit improved emulsifying properties and increased dispersibility in aqueous systems, making them suitable for a wide range of applications including food, pharmaceuticals, and cosmetics [9]. The modification processes, such as hydroxylation, enzymatic hydrolysis, acetylation, and defatting, result in lecithins with specific enhanced hydrophilicity and oil-in-water emulsifying properties [6,7,10,11].

In the food industry, lecithin is widely used for its emulsifying properties. It helps in blending ingredients that typically do not mix, like oil and water. It is commonly used in products like mayonnaise, salad dressings, chocolate, and baked goods [12,13]. Lecithin supplements are often promoted for their potential health benefits. These include improving cholesterol levels, serving as a source of choline (important for liver function, nerve function, and muscle movement), and possibly aiding in the treatment of certain neurological and cognitive disorders [14]. 

Beyond food, lecithin is used in pharmaceuticals as a dispersing agent, in cosmetics for its emollient and skin-softening properties, and even in paints and other industrial applications for its stabilizing and emulsifying abilities. While generally recognized as safe, lecithin sourced from soy and eggs can cause allergic reactions in individuals sensitive to these substances [3]. Commercial lecithin comes in several forms, including granules, liquids, and powders, each suited for different applications. Lecithin is a good source of essential fatty acids and contains various beneficial compounds like phosphatidylinositol and phosphatidylserine. In summary, lecithin is a versatile and widely used ingredient known for its emulsifying properties, with applications ranging from food products to pharmaceuticals and industrial uses [15].

## 2. Structure-Forming Features of Lecithin

From a material science perspective, lecithin is valued for its unique physicochemical properties, which make it an essential ingredient in various formulations, ranging from food to pharmaceuticals, and its multifunctionality is highly valued [16]. Its capacity to form and stabilize emulsions, alter rheological properties, and interact with various molecules makes it a versatile tool in developing complex material systems, particularly in food science, pharmaceutical formulations, and cosmetic products [17,18]. The crucial structure-forming features are listed in Table 1.

**Figure 1:**
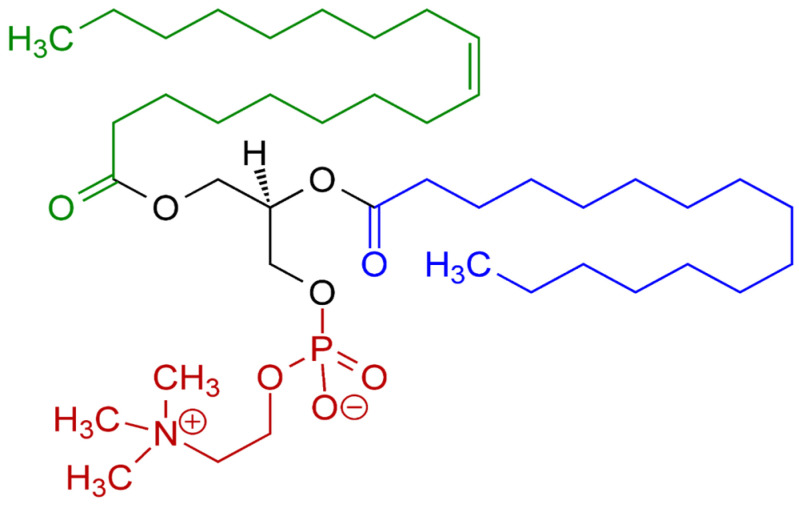
Phosphatidylcholine, the most common type of phospholipid in lecithin. Red—choline residue and phosphate group; black—glycerol residue; green—monounsaturated fatty acid residue; blue—saturated fatty acid residue.

Lecithins from different origins can vary significantly in their physicochemical properties and functionalities. The source of the lecithin largely influences its fatty acid composition, phospholipids like phosphatidylcholine (PC—Figure 1), phosphatidylethanolamine (PE), and phosphatidylinositol (PI), and other minor components, which, in turn, affect its behavior and performance in various applications [3,15,16]. 

Some key differences based on the origin of lecithin include chemical content variability, encompassing variations in fatty acid composition, phospholipid content, purity and impurities, and related allergenic potential or nutritional aspects, as well as resultant rheological properties, emulsifying ability, or temperature stability [3]. Specifically, the type of fatty acids present in lecithin can differ based on its source. The most used soy lecithin has different fatty acid types and ratios [19] compared to egg yolk [20] or sunflower lecithin [21]. This variation impacts properties such as melting point, viscosity, and emulsifying efficiency. For example, egg yolk lecithin typically has a higher content of PC compared to soy lecithin. The phospholipid composition affects the amphiphilic nature and the emulsifying properties of the lecithin [16,19,20,21].

The extraction and processing methods used lead to changes in purity and contamination level which affect the functional properties of the lecithin, such as its taste, color, and odor, and are particularly important in food applications. Both the source of lecithin and the extraction method can also be important from an allergenicity perspective. Soy lecithin is a concern for individuals with soy allergies, whereas sunflower or egg lecithin might be preferred alternatives. Considering the nutritional profile, including the type and ratio of essential fatty acids, the source of lecithin regulates its use in dietary supplements and health products [3].

The differences in the molecular organization of lecithins lead to observed variations in their impact on the rheology of the systems they are used in, like viscosity-modifying effects and gelation behavior. The efficiency and stability of emulsions formed with lecithins vary due to differences in their surface-active properties [3,12,13,14,15]. Varying thermal stability impacts applications involving heating or cooling processes, highlighting the adequacy of the origin source of lecithin as an important factor. Such variability confirms the importance of selecting the right type of lecithin for specific applications, considering not only its functional properties but also factors like nutritional requirements, potential allergenicity, and sensory attributes [3,12,13,14,15,16,17]. Understanding the source-specific characteristics of lecithin allows for more precise formulation and optimization in various industrial applications. In food science, the most extensively studied sources of lecithin are primarily soybeans, eggs, and, to a lesser extent, sunflower [19,20,21]. 

## 3. Oleogelation Process and Methods

Lecithin can play several important roles in the formation and stabilization of oleogels, capitalizing on its unique amphiphilic nature and emulsifying properties. 

Oleogelation, a process that entails the conversion of liquid oils into semi-solid or solid gels through the incorporation of structuring agents or oleogelators, is a key aspect of food science. Oleogelation, at its core, is based on the molecular self-organization of oleogelating compounds in the liquid oil phase, yielding a three-dimensional gel matrix [22].

Oleogels could offer a solution to mitigate the harmful health effects associated with the excessive consumption of saturated and trans fats, resulting in their reduction while maintaining the sensory and textural attributes of food products [23]. Oleogels impart a superior, creamier, and smoother texture to a variety of food products, including spreads, baked goods, and confectionery, increasing their sensory appeal and consumer acceptance [24]. Oleogels impart stability to oil-based ingredients, effectively preventing phase separation and oxidation, thereby extending the shelf life of food products—an attribute of great commercial and consumer value [25]. The ability to use natural oleogelators ensures compliance with the emerging consumer preference for clean and recognizable ingredient labels, supporting the link between modern food technology and clean labeling trends [26]. Oleogelation provides fertile ground for the development of innovative food products, examples of which include structured emulsions, reduced-fat products, and novel snacks.

**Figure 2:**
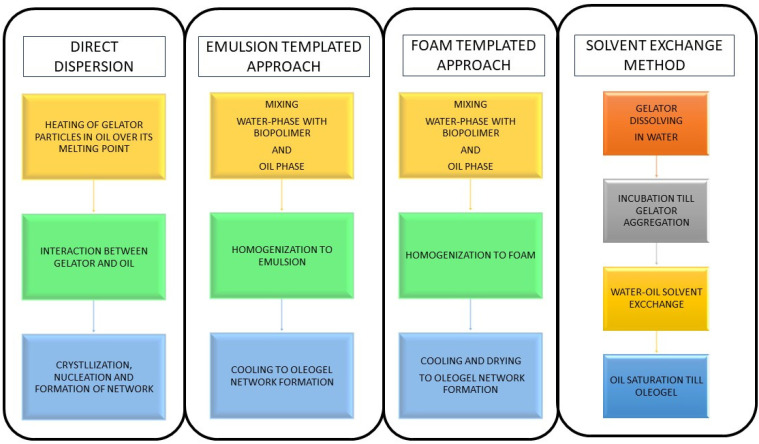
Oleogelation method templates.

A number of methods are used for oleogelation (Figure 2) depending on the physico-chemical properties of the oleogelator and the requirements of the final product. Among these, solvent exchange, cooling and crystallization, high-pressure homogenization, physical mixing, and emulsification are the most commonly used [27]. A solvent exchange technique in which the oleoelator is dissolved in a solvent miscible with the oil phase is used. After cooling, the solvent is meticulously removed, leaving a stable gel network [23,24,25,26]. Some oleogelators typical of waxes participate in the oleogelation process by crystallizing at temperatures below their respective melting points, thereby coalescing to form a gel matrix [27]. High-pressure homogenization is used to break up the oleogelator particles, thereby reassembling them into a coherent gel structure—an industrially relevant method for various oleogelating applications [28]. Mechanized mixing and the application of shear forces are used to disperse oleogelators in the oil phase, nurturing their final gelation after cooling. Oleogelation by emulsification entails the formation of an oil-in-water emulsion, with the aqueous phase containing the oleogelator. The subsequent removal of water culminates in the appearance of a gel structure in the oil phase [22].

The choice of oleogelator depends on the specific application and the desired attributes of the oleogel. Major oleogelators include hydrocolloids, polymers, waxes, fatty acids and monoglycerides, microcrystalline cellulose, gellan gum, agar, xanthan gum, and so on. They take on the mantle of structuring agents, absorbing water and trapping oil droplets, giving oleogels texture and stability. Cellulose derivatives, starch derivatives, and pectin—examples of polymers—are used as oleogelators. Their usefulness stems from their propensity to form gel networks through oil-phase interactions. Natural and synthetic waxes such as beeswax, candelilla wax, and hydrogenated vegetable oils play an important role in oleogelation. These compounds solidify upon cooling, providing stability and improving the texture of oleogel matrices. Saturated fatty acids and monoglycerides, distinguished by their crystalline properties, act as oleogelators, contributing to the formation of structural fats integral to bakery and confectionery recipes. The use of microcrystalline cellulose as an oleogelator is important in selected applications, especially in low-fat spreads and salad dressings, where its molecular nature helps form a gel network [26].

As the global food industry navigates the evolving landscape of health-conscious consumers, oleogelation is poised to advance its position as a key technology for developing healthier and more sustainable food products [22].

## 4. Lecitin’s Functions in Oleogelation

As the structure–function relation is fundamental in oleogelation and oleogels, lecithin usability can be assessed within five different criteria: 1. oleogelation facilitator; 2. structure-forming impact; 3. texturing agent; 4. functionality provider; and 5. nutrition improver.

### 4.1. Facilitation of Oleogel Formation

Lecithin, due to its natural and versatile properties, shows different functionalities, such as acting as an emulsifier and co-oleogelator in the oleogel system. Functionalities according to the structure function criteria in oleogels are grouped in Table 2.

#### 4.1.1. Oleogelator

Different fractions of soy lecithin, specifically the ethanol-soluble fraction, phospholipid fraction, and glycolipid fraction, were evaluated and found to function as oleogelators, forming oleogels at varying concentrations. The ethanol-soluble fraction and phospholipid fraction formed oleogels at 30% (wt%), while the glycolipid fraction formed oleogels at 15% [29]. Lecithin was also proved to act as a co-oleogelator in the hybrid oleogel system, influencing the structure of the host oleogelator and the formation of self-sustained structures. Okuro et al. (2018) [30] demonstrated the behavior of soybean lecithin in a hybrid oleogel system formed by β-sitosterol and γ-oryzanol. Authors have noted that addressing the role of the co-oleogelator can provide the opportunity to tune soft materials with desired properties.

#### 4.1.2. Emulsifier

Lecithin acts as a powerful emulsifier in oil-continuous emulsions, adsorbing to fat crystals at the triglyceride oil/water interface and making their surface more polar, which was observed by an increase in the contact angle measured through the oil at the fat crystal/oil/water interface [31]. Lecithin enables gel formation even below the critical gelling concentration of fruit wax, and it alters the molecular assembly properties of the fruit wax due to the interactions between the polar moieties of the oleogelators [32]. Authors have hypothesized that this happens in those interactions, which consequently impacts the hydrophobic tail (re)arrangement in gelator–gelator and solvent–gelator interactions, as both the oil-binding capacity and the thixotropic recovery were observed to be enhanced upon lecithin addition in [33].

Soy lecithin has been used in the formation of lecithin-based oleogels (LOGs) and lecithin-based oleogel emulsions (LOGEs), where it forms reverse worm-like micelles that entangle to create the structure of the LOG. In LOGEs, it interacts synergistically with stearic acid (SA) bilayers to stabilize the three-dimensional network. The results indicate that LOGs were primarily formed through the entanglement of bundles of reverse worm-like micelles of SL [33].

Lecithin acts as an emulsifier and stabilizer in oleogels and oleogel emulsions, impacting various physicochemical properties. Sahu et al. (2021) reported how two different lecithins such as sunflower lecithin and soya lecithin impact on the various physicochemical properties of candelilla wax and rice bran oil oleogels. The impact was both lecithin dose dependent and origin dependent [34]. 

Lecithin acts as an emulsifier in oleogels, helping to stabilize the mixture by reducing the surface tension between the oil and other ingredients. This results in a more uniform and stable product. Dhal et al. (2023) [35] reported that a sunflower lecithin-containing oleogel sample had a small grainy morphology of the wax crystals which seemed well distributed throughout. Another study found that at a critical soy lecithin content, the oleogel showed a stable and repeatable wax network structure. Sena et al. (2022) showed that the addition of soy lecithin can modify the microstructural and physicochemical properties of soybean oil- and soy wax-based oleogels, and the obtained structures, containing 5 mg of soy lecithin, showed dense crystal microarchitectures with good mechanical and thermal properties [36].

### 4.2. Structure-Forming Impact

In the context of oleogels, structure-forming refers to the organization and arrangement of the components within the gel system. Oleogels are gels formed by structuring edible oils or fats with the help of gelling agents. These gelling agents are responsible for creating a three-dimensional network or structure within the oil phase, which imparts the desired texture and functional properties to the oleogel. The structure of an oleogel is crucial because it determines its physical characteristics, such as its texture, stability, and suitability for various applications.

#### 4.2.1. Structural Organization Facilitator

The lecithin used, as well as its solvents, has a strong impact on structural arrangement, as revealed by small-angle X-ray diffraction. Bodennec et al. (2016) [37] reported a reverse hexagonal (HII) lattice structure with a d-spacing of 52 A and liquid crystallites ∼1200 A in length that rearranged with temperature evolving towards a less-ordered isotropic fluid unable to gel in lecithin/canola oil oleogels. Also, Shchipunov and E. V. Shumilina (1995) [38] studied a series of polar solvents in order to reveal those capable of producing a thickening effect on hydrocarbon solutions of lecithin as they observed that the difference between gel-forming and non-gel-forming polar solvents is caused by their orientation and localization in the polar moiety of a lecithin molecule.

#### 4.2.2. Structural Modifier

Okuro et al. (2018) [30] studied a double system of β-sitosterol and γ-oryzanol and reported that its intermolecular complex formation is hindered by lecithin. The authors observed that the addition of lecithin causes changes in the typical fibril aggregation of BG, promoting the suppression of ribbons to some extent. The same authors also suggested that lecithin alters the molecular assembly properties of the fruit wax due to the interactions between the polar moieties of the oleogelators as they observed the effect of lecithin on the crystallization and gelation of fruit wax with sunflower oil.

#### 4.2.3. Microstructural Formation

At the microscale, gels showed a 3D network composed of microfibers with an average diameter of ∼1 μm. The proposed self-assembly mechanism was based on the packing of reverse hexagonal tubules parallel to the axis of the fibers [37]. The gel network is formed due to the branching and overlapping of these bundles at the junction zones along the reverse micellar chains [30,35]

Okuro et al. (2018) reported that microscopy images show that the typical fibril aggregation of β-sitosterol and γ-oryzanol changed when lecithin was added, promoting, to a certain extent, the suppression of phytosterols ribbons formation. Small-angle X-ray scattering showed that the formed nanostructures (building blocks) were dependent on the type of solvent and the β-sitosterol and γ-oryzanol/lecithin ratio in the mixture of oleogelators [30]. 

#### 4.2.4. Crystal Characteristics Modifier

Lecithin has also been reported to have a role in crystallization, a key determinant of the final properties of the organogel networks. Ghan et al. (2020) observed this for soy lecithin in the solidification of palm oil [39]. 

Lecithin has the potential to alter the shape and size of fat crystals by interacting with wax molecules, resulting in better crystal packing and fewer crystal defects. Sahu et al. (2021) reported that in the case of candelilla wax usage, the structural reorganization and crystal growth due to the addition of lecithin affected the oleogel’s mechanical property significantly [35]. Also, Okuro et al. (2018) confirmed that the lecithin phospholipids acted as a crystal habit modifier, changing the microstructure of the oleogel, as observed by polarized light microscopy [32].

#### 4.2.5. Self-Assembly Promoter

Lecithin promotes the self-assembly of molecules via non-covalent interactions, as indicated by thermal and rheological measurements, as shown by Okuro et al. (2019) [30]. As it has been known for modifying the interfacial tension relationships in the fat and oil industry from the very beginning [10], its impact on structural characteristics like firmness, the gel–sol transition, and the melting temperatures of organogels seem obvious. Specifically, the fact that, due to its amphiphilic nature, lecithin might coexist as a different phase in the oleogel system causes structural changes in the gel network.

#### 4.2.6. Thermal Behavior Changer

Among the different characteristics of oleogels, lecithin affects the thermal behavior of the structure by delaying both crystallization and gel formation [30]. Therefore, the stability against the temperature of the oleogel system is influenced by the presence of lecithin [32]. Sena et al. (2022) reported that material characteristics such as the thickness, length, and density of the wax crystals (needle-shaped) varied as the soy lecithin content was changed [36]. However, Bodennec (2016), through using rheological tests, observed that lecithin/canola oil oleogels transitioned to liquids at 50–55 °C regardless of lecithin concentration (10, 20, and 30 wt%) [37]. This confirms the previous conclusions regarding the co-gelator action of lecithin, which enhances some features in certain mixtures, but lecithin does not generally act as a thermal behavior modifier.

#### 4.2.7. Formation of Hydrogen-Bonded Networks

It was previously assumed that in the presence of polar substances, lecithin contributes to the formation of hydrogen-bonded three-dimensional networks [40]. In the organogel model, lecithin’s phosphate group attaches to both gel-forming and non-gel-forming solvent molecules via hydrogen bonds, influencing the mobility of the choline residue [31]. Ghan et al. (2020) observed soy lecithin’s contribution to intermolecular hydrogen bonding and van der Waals forces for self-aggregation in palm olein [29]. The specific behavior of lecithin was observed by Y. Shchipunov and E. Shumilina (1996) [40], who suggested that in the proposed model of cylindrical reverse micelles, lecithin molecules are bridged by solvent molecules, forming hydrogen-bonded linear networks.

#### 4.2.8. Modifier of Hydrogel Structure

Lecithin can alter the inner hydrogel’s structure through its self-assembly and therefore its transport properties. Transport properties are one of the most crucial assets of hydrogel samples, influencing their main application potential [41]. In xerogel samples, when lecithin was added, the ability to influence transport properties was better observed as it decreased the values of the diffusion coefficient independently of the dye used and the type of crosslinking [41]. Tamura and Ichikawa (1997) reported that intermolecular 1:1 complexes were formed between lecithin and 12-HSA, which caused a structural change in the fibrous network in the 12-HSA organogel and consequently induced gel deformation [42]. Also, Ghan et al. (2020) observed that soy lecithin influences the formation of β’-type polymorphic structures in palm olein-based organogels and formed rod-shaped tubules in palm olein-based organogels [39].

### 4.3. Texturing Agent

Texture, in the context of oleogels, refers to the physical characteristics and sensory properties of the gel, specifically how it feels and behaves when touched, manipulated, or consumed. The texture of an oleogel is a critical aspect as it determines the mouthfeel, spreadability, and overall sensory experience when used in various food products. Texture can be controlled and modified to achieve specific desired properties in oleogels. 

#### 4.3.1. Texture and Structural Modifier

Lecithin modifies the texture and structure of oleogels, improving spreadability and oil-binding capacity [29,30,31,32,33,34,35,36,42]. Specifically, Okuro et al. (2018) reported that lecithin (LEC) increases gel hardness and has a synergistic effect on gel strength when combined with fruit wax (FW) [32]. A synergistic effect on gel strength was observed at FW/LEC ratios of 75:25 and 50:50 compared to the corresponding single-component formulations (100:0 and 0:100). Also, Aguilar-Zárate et al. (2019) [43] observed that the addition of 1% (*w*/*w*) soy lecithin increased the shear moduli 10-fold and gel hardness 20-fold for 10% ethylcellulose (EC) oleogels, while large-amplitude oscillatory shear rheology demonstrated similar solid-to-fluid transitions, indicating that the polymer drives elastic softening and network failure. 

The addition of unsaturated lecithin to EC oleogels promoted a more gradual thickening response compared to gels containing saturated lecithin or only EC [43]. However, the incorporation of lecithin in the combination of β-sitosterol and γ-oryzanol and SFO resulted in harder oleogels [30], as was the case for 10% ethylcellulose (EC) oleogels, where the addition of 1% (*w*/*w*) soy lecithin increased the shear moduli 10-fold and gel hardness 20-fold [43]. Similar trends were observed for the penetration force of the gels [43]. The hardness of the oleogels and oleogel emulsions (LOGs and LOGEs) increased with an increase in stearic acid (SA). In samples containing both soy lecithin (SL) and SA, the LOGEs were harder than the LOGs, indicating that lecithin contributes to the texture of the oleogels [34]. Structural reorganization and crystal growth due to the addition of lecithin significantly affect the oleogel’s mechanical properties, improving its spreadability. Emulsifiers such as sunflower lecithin do not affect the firmness and elastic component of oleogels, according to stress relaxation studies. Varying the concentrations of soy lecithin (SYL) in oleogels results in a soft texture [36]. The ESF resulted in an oleogel with a harder and less cohesive texture than the PLF-supported oleogel. The GLF at 20% formed an oleogel with better texture characteristics in terms of hardness [29].

#### 4.3.2. Inducer of a Gel-like State

Lecithin can induce the transition of non-aqueous solutions to a gel-like state when combined with various polar substances [38,44]. Even below the critical gelling concentration (Cg) of fruit wax, the addition of lecithin enabled gel formation in [32]. Lecithin plays a crucial role in the formation of oleogels. The range of wo values (molar ratio of [H_2_O]/[lecithin]) leading to gel formation at high lecithin concentration (30 wt%) was broader than at low concentration (10 wt%) in [37]. Lecithin, in the presence of certain polar solvents like glycerol, formamide, ethylene glycol, and water, can induce the formation of organogels [38].

#### 4.3.3. Oil Capacity Enhancer

Lecithin is a substance that enhances the oil-holding and oil-binding capacity of oleogels [29,30]. Soya lecithin (SL) and glyceryl monostearate (GMS) have high oil-binding capacities in palm olein-based organogels [29].

### 4.4. Functionality Provider

For oleogels, functionality refers to the specific roles and capabilities that oleogels provide when used as ingredients or components in food and non-food applications. Oleogels are valued for their ability to modify the texture, structure, and other properties of products, and their functionality can vary depending on the intended use. 

Lecithin is used in various applications, including as an oleomargarine and in shortenings, confections, coatings and icings, and vitamin oils. Delaying crystallization and gel formation can affect the thermal behavior of oleogels, as such a modification results in the extension of their shelf lives. 

#### 4.4.1. Shelf Life Extender

The inclusion of lecithin could extend the shelf lives of oleogels by affecting their melting points [35] and by preventing the separation of ingredients and maintaining the product’s stability over time [31,32]. Therefore, it improves the temperature stability of oleogel systems against temperature changes [46].

#### 4.4.2. Resembles Edible Fat

The thickening response of EC oleogels containing unsaturated lecithin more closely resembles that of a model edible fat (lard) [43]. The incorporation of SYL can impact the color of the oleogels, making them slightly yellowish [36]. The addition of unsaturated lecithin to EC oleogels promoted a more gradual thickening response compared to gels containing saturated lecithin or only EC. Lecithin is used in the fat and oil industry to prevent rancidification [10]. The water mobility restriction capacity can be improved to retain moisture in oleogels, preventing them from drying out and maintaining their consistency over time [36]. In food-grade oleogels, lecithin can improve the taste by masking any unpleasant flavors from other ingredients. Lecithin can improve the viscosity of oleogels, making them thicker and more stable. This can enhance the user’s experience, particularly in cosmetic and food applications. Lecithin can improve the dispersion of ingredients in oleogels, preventing clumping and ensuring a smooth, uniform product. Lecithin can enhance the solubility of certain ingredients in oleogels, ensuring they are evenly distributed and fully integrated into the product. Lecithin is a cost-effective ingredient for oleogels. It performs multiple roles, reducing the need for other, potentially more expensive, ingredients.

### 4.5. Nutrition

Nutritive values, bioavailability, and bioaccessibility are related in oleogels, particularly when considering their impact on the nutritional profile and health benefits of the foods or supplements in which they are used. Nutritive values refer to the nutritional content of a food or product, including the presence of macronutrients (such as fats, proteins, and carbohydrates) and micronutrients (such as vitamins and minerals). Oleogels are often used to structure and deliver edible oils and fats in various food products. The nutritive values of these products are influenced by the type of oil or fat used in the oleogel and its nutritional composition. For example, if a healthier oil with a favorable fatty acid profile (e.g., high in unsaturated fats and low in saturated fats) is used in an oleogel, it can contribute positively to the nutritive values of the final product by reducing saturated fat content and increasing the presence of beneficial unsaturated fats.

Bioavailability refers to the extent and rate at which a nutrient is absorbed and becomes available for use or storage in the body after consumption. It is influenced by various factors, including the form in which the nutrient is presented in the food matrix. In the case of oleogels, the structured nature of the oil or fat can impact the bioavailability of certain fat-soluble nutrients (e.g., vitamins A, D, E, and K) present in the product. Oleogels can potentially enhance the bioavailability of these nutrients because they may facilitate their dispersion and absorption in the digestive system. The controlled release of lipids from oleogels during digestion can also affect the bioavailability of certain bioactive compounds.

Bioaccessibility refers to the proportion of a nutrient that is released from the food matrix and becomes available for absorption during digestion. It represents the initial stage of the release of nutrients from the food. Oleogels can influence the bioaccessibility of nutrients because they can act as carriers or delivery systems. The gel structure can protect sensitive nutrients from degradation during processing and storage, and it can control their release in the gastrointestinal tract, impacting their bioaccessibility. For example, oleogels can encapsulate and protect lipophilic bioactive compounds such as phytochemicals or nutraceuticals, improving their stability and bioaccessibility. 

#### 4.5.1. Natural Ingredient

Lecithin is a natural ingredient, which can be a selling point for oleogels intended for health-conscious consumers. It is often preferred over synthetic alternatives. Lecithin can act as a carrier for nutrients in oleogels, helping to distribute them evenly throughout the product. Lecithin’s adsorption to fat crystals and the oil/water interface may facilitate the transport of certain nutrients, as suggested by the mention of phospholipid composition in [31,32,33].

#### 4.5.2. Antioxidant Properties

Lecithin has antioxidant properties which can help protect the other ingredients in oleogels from oxidation, thereby maintaining their quality and effectiveness. Lecithin, when added to the natural antioxidants quercetin, dihydroquercetin, and α-tocopherol, was found to decrease the antioxidant effectiveness of flavonoids during the initiated and autoxidation of methyl oleat. The effect value increased with an increase in the lecithin concentration [46]. In mixtures of α-tocopherol and lecithin, the latter did not influence the tocopherol antioxidant effectiveness (additivity) or lead to an increase in the inhibition effectiveness (synergism) [46]. The physical and microstructural characteristics of the LOGEs, including lecithin, delay the oxidation rate of the systems by preventing interactions between lecithin and other molecules prone to oxidation [34].

#### 4.5.3. Enhancing Bioavailability and Bioaccesibility

As slow crystallization kinetics is correlated with better crystal packing and fewer crystal defects, lecithin can potentially enhance the bioavailability of the oleogels [35]. 

Lecithin can enhance the bioavailability of certain nutrients in oleogels, as well as their solubility and slow crystallization kinetics, making them more easily absorbed by the body. The inclusion of lecithin in oleogels enhances their properties, allowing them to be included in various food products.

## 5. Conclusions

Oleogels can significantly influence the nutritional value, bioavailability, and bioaccessibility of nutrients in food products, with their structure, composition, and digestive behavior affecting nutrient release and absorption. This has important implications for the nutritional quality and health benefits of food and supplements. Lecithin, serving as an effective emulsifier, plays a crucial role in stabilizing oleogels, especially those containing water or aqueous solutions. It prevents phase separation, enhances stability, and can contribute to gel network formation, interacting with other oleogelators to immobilize the oil phase and improve the gel’s consistency. Lecithin’s impact extends to modifying oleogels’ rheological properties, such as their viscosity, elasticity, and spreadability, which are vital for texture and mouthfeel in food applications.

In pharmaceutical and nutraceutical contexts, lecithin improves the bioavailability of active ingredients through micelle and liposome formation, facilitating controlled release. It also influences lipid crystallization in oleogels, affecting texture and stability by altering crystal size and distribution. Synergistically, lecithin enhances gelation and mechanical strength when combined with other oleogelators like waxes or polymers and aids in ingredient dispersion and homogenization for uniform oleogels. Additionally, in oleogel-based coatings, it contributes to barrier properties, improving preservation. Lecithin also adds nutritional value, serving as a source of phospholipids and choline. The effectiveness of lecithin in oleogels depends on its concentration, the type of oil used, and the presence of other ingredients, highlighting the importance of formulation optimization to fully leverage lecithin’s benefits in oleogel applications.

## Figures and Tables

**Table 1:** The fundamental structure-forming features of lecithin for oleogel formulations.

Feature	Description
Amphiphilic nature	Amphiphilic nature, meaning having both hydrophilic and lipophilic parts. This characteristic is fundamental to its role as an emulsifier, allowing it to stabilize mixtures of oil and water by reducing the surface tension at the interface.
Surface activity	Lecithin adsorbs at oil/water interfaces, forming a monolayer or bilayer, stabilizing emulsions against coalescence. This surface activity is crucial in the creation of stable emulsions, micelles, and liposomes.
Rheological modifier	Lecithin modifies the rheological properties of substances, acting as a viscosity enhancer or stabilizer that affects the flow behavior of liquids and semi-solids.
Phase behavior and self-assembly	Lecithin molecules can self-assemble into various structures, such as micelles, bilayers, and vesicles, depending on the concentration, temperature, and the presence of other substances. This self-assembly behavior is critical in the formation of liposomes for drug delivery and other nano-scale applications.
Compatibility with other molecules	Lecithin interacts with other molecules like proteins, polysaccharides, and other surfactants, which can be used to tailor the properties of emulsions, coatings, and encapsulation systems.
Thermal properties	Lecithin’s behavior under different temperature conditions, including its melting point and thermal stability, is important for applications that require thermal processing.
Solubility and dispersibility	Lecithin’s solubility in various solvents (water, oils, organic solvents) and coordination of insoluble compounds make it suitable for a wide range of applications.
Barrier properties	In coatings and films, lecithin can contribute to moisture, gas, and lipid barrier properties, which is significant for food preservation and packaging.
Biocompatibility and biodegradability	Lecithin is biocompatible and biodegradable, making it suitable for applications in food, pharmaceuticals, and cosmetics, where non-toxicity and environmental friendliness are crucial.

**Table 2:** Lecithin functionalities in oleogels.

Relation Criteria	Functionalities	Ref.
Oleogelation facilitator	Oleogelator, co-oleogelator, emulsifier.	[29,30,31,32,33,34,35,36]
Structure-forming impact	Structural organization facilitator, structural modifier, microstructural formation, crystal characteristics modifier, self-assembly promoter, thermal behavior changer, formation of hydrogen-bonded networks, modifier of hydrogel structure.	[10,29,30,31,32,33,34,35,36,37,38,39,40]
Texturing agent	Texture and structural modifier, inducer of gel-like state, oil capacity enhancer.	[29,30,31,32,33,34,35,36,37,38,39,40,41,42,43]
Functionality provider	Shelf life extender, resembles edible fat.	[10,31,32,35,36,43,44]
Nutrition improver	Natural ingredient, antioxidant properties, enhancing bioavailability and bioaccessibility.	[31,32,33,34,35,45]

## Data Availability

Not applicable.
